# Multisensory temporal processing in schizophrenia and bipolar disorder: implications for psychosis

**DOI:** 10.1038/s41537-024-00502-z

**Published:** 2024-10-30

**Authors:** Maria Bianca Amadeo, Andrea Escelsior, Davide Esposito, Alberto Inuggi, Silvio Versaggi, Giacomo Marenco, Yara Massalha, Jessica Bertolasi, Beatriz Pereira da Silva, Mario Amore, Gianluca Serafini, Monica Gori

**Affiliations:** 1 U-VIP Unit for Visually Impaired People, Fondazione Istituto Italiano di Tecnologia, Genoa, Italy; 2 IRCCS Ospedale Policlinico San Martino, Genoa, Italy; 3 Department of Neuroscience, Rehabilitation, Ophthalmology, Genetics, Maternal and Child Health (DINOGMI), Section of Psychiatry, University of Genoa, Genoa, Italy; 4 Department of Pathophysiology and Transplantation, University of Milan, Milan, Italy; 5 Department of Informatics, Bioengineering, Robotics, Systems Engineering (DIBRIS), University of Genova, Genova, Italy

**Keywords:** Psychosis, Neuroscience

## Abstract

Structuring sensory events in time is essential for interacting with the environment and producing adaptive behaviors. Over the past years, the microstructure of temporality received increasing attention, recognized as a fundamental factor influencing cognitive, affective, and social abilities, whose alteration can underlie the etiopathogeneses of some clinical symptoms in psychiatric disorders. The present research investigated multisensory temporal processing in individuals with schizophrenia (*N* = 21), bipolar disorder (*N* = 20) and healthy controls (*N* = 21) in order to explore a plausible link between multisensory alterations in the temporal order of events and the psychopathological dimensions underlying psychosis. We asked participants to temporally order audio-tactile, visual-tactile, and audio-visual stimuli, and we administered different psychopathological scales to explore depressive, manic and psychotic symptoms. Results demonstrated that both subjects with schizophrenia and bipolar disorder are less precise in temporal order judgment independently of the sensory modalities involved. Interestingly, reduced precision in temporal processing of patients is positively associated with the presence and severity of positive symptoms. Our findings support the hypothesis that low-level sensory alterations in temporal structure may contribute to the emergence of clinical symptoms such as delusions, hallucinations, and disorganized behaviors.

## Introduction

Sensory processing is the neurobiological mechanism through which the nervous system decodes environmental sensory inputs from diverse modalities, playing a vital role in self-perception and interaction with our surroundings. Our brain processes a multitude of sensory information every moment, integrating, separating, and ordering it in space and time to derive a coherent and unified understanding of the environment. In recent years, the microstructure of temporality (i.e., how we process sensory events in time) has received increasing attention in psychiatry. It has been considered a fundamental element of consciousness itself whose alteration can underlie the etiopathogenesis of various clinical symptoms^1–4^.

Classic phenomenological investigations of psychiatric disorders have documented numerous examples of abnormal time experiences^5–8^, suggesting that the disintegration of time clearly has profound psychopathological effects on how one perceives oneself, the sensory world, and interacts with others^9^. Despite these findings, most experimental studies have focused on the subjective experience of time durations, rather than on how patients encode time events^10^. The links between the sensory construction of temporal structure and clinical symptoms remain debated.

Research has demonstrated that failure to correctly detect sensory (a)synchrony or identify temporal order is associated with impairments in cognitive, affective, and social abilities^11^. Among the psychiatric disorders investigated, schizophrenia (SZ) has received the most attention. It has been shown that patients with SZ are more likely to integrate temporally separate stimuli in the visual^12–17^, audio-visual^18–21^, and audio-tactile^22^ domains. In contrast, only one study investigated temporal order processing in patients with bipolar disorder (BD), showing that their performance in temporally ordering visual stimuli was between that of SZ patients and controls^12^. Few studies explored the association between temporal structure and psychotic symptoms, though some suggest a potential link (for a review on SZ patients, see ref. ^23^). Altered low-level temporal processing in SZ patients has been linked with disorganization symptoms^13,24,25^ and hallucinations^18^. Accordingly, healthy individuals with high schizotypal traits levels have been shown to have lower tactile temporal resolution^26^ and extended tolerance to audio-tactile temporal asynchronies^27^.

While distinct impairments in temporal structure within SZ have been well-documented, there remains a lack of comprehensive studies investigating the association between the temporal processing of multisensory events and key psychopathological dimensions underlying psychosis. If this link actually exists, this would open important perspectives for the screening, assessment and rehabilitation of psychosis, where temporal processing and sensory training could be exploited^28^.

Based on these premises, here we aimed to deepen the role of structuring sensory events in time in mental disorders and its implications in psychopathology. Using a transdiagnostic approach, we hypothesized that alterations in temporal order of events would be specifically associated with positive psychotic symptomatology rather than a particular diagnosis. Hence, we assessed audio-tactile, audio-visual, and visuo-tactile temporal order skills in patients with SZ, BD, and healthy controls (HC) while administering different psychopathological scales to explore depressive, manic, and psychotic symptoms underlying the diseases. Multisensory stimuli were selected based on evidence suggesting differences between unisensory and multisensory perception in patients^29,30^ and impairments in multisensory temporal acuity extending beyond unisensory deficits in SZ^18^, indicating specificity of multisensory inputs. Additionally, investigating temporal order of different pairs of multisensory stimuli allowed us to explore whether the deficit can be generalized and is independent of the sensory system involved.

## Results

**Fig. 1:**
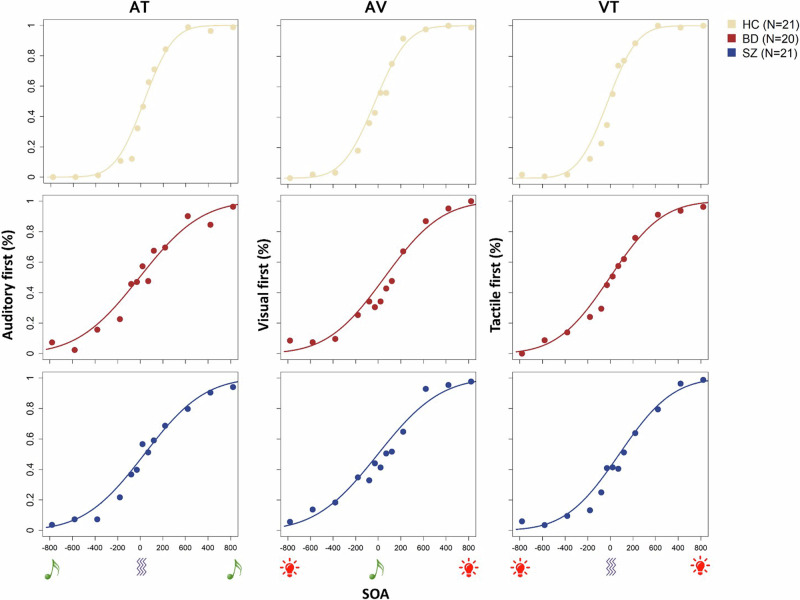
Averaged results of HC (first row), BD patients (second row) and SZ patients (third row) for each condition (AT, AV and VT) of the TOJ task. Left: Proportion of trials (averaged across participants) judged as “Auditory stimulus delivered before the tactile stimulus” plotted against the stimulus onset asynchrony (SOA) between auditory and tactile stimuli (0 ms corresponds to synchrony; negative and positive values correspond to auditory stimulus presented before and after the tactile stimulus respectively). Center: Proportion of trials (averaged across participants) judged as “Visual stimulus delivered before the auditory stimulus” plotted against the SOA between visual and auditory stimuli (0 ms corresponds to synchrony; negative and positive values correspond to visual stimulus presented before and after the auditory stimulus respectively). Right: Proportion of trials (averaged across participants) judged as “Visual stimulus delivered before the tactile stimulus” plotted against the SOA between tactile and visual stimuli (0 ms corresponds to synchrony; negative and positive values correspond to visual stimulus presented before and after the tactile stimulus respectively). Data are fitted with the Gaussian error function.

21 patients with Schizophrenia (SZ), 20 patients with Bipolar Disorder (BD) and 21 healthy controls (HC) performed a Temporal Order Judgment task (TOJ). They were presented with pairs of audio-tactile (AT), visuo-tactile (VT) or audio-visual (AV) stimuli with different stimulus onset asynchronies (SOAs) and were instructed to report which stimulus appeared first. The mean number of trials for each condition and group was: HC-AV = 51.81, HC-AT = 51.76, HC-VT = 52, BD-AV = 52, BD-AT = 52, BD-VT = 51.55, SZ-AV = 52, SZ-AT = 51.29, SZ-VT = 51.52. Figure 1 shows the averaged psychometric functions for each group and condition.

**Fig. 2:**
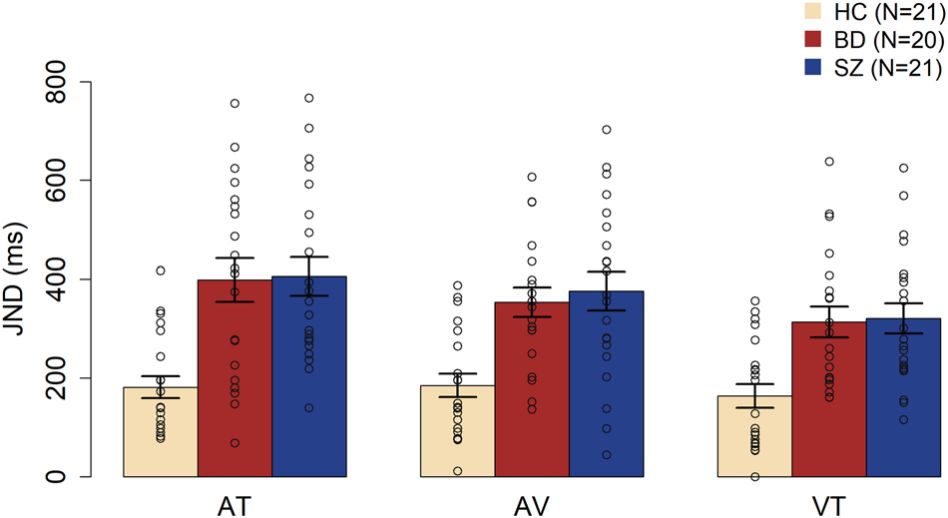
JNDs (mean ± standard error of the mean – SEM) of healthy controls (HC), patients with Bipolar Disorder (BD) and Schizophrenia (SZ) in the audio-tactile (AT), audio-visual (AV) and visuo-tactile (AT) condition of the temporal order judgment task (TOJ). Dots represent individual data.

Statistical analyses demonstrated worse temporal precision of both SZ and BD patients compared with HC in all conditions (Fig. 2). Data were normally distributed for some (for BD-AT: W = 0.96, *p* = 0.6; for SZ-AT: W = 0.92, *p* = 0.1; for HC-AV: W = 0.93, *p* = 0.2; for BD-AV: W = 0.97, *p* = 0.8; for SZ-AV: W = 0.98, *p* = 0.9; for SZ-VT: W = 0.94, *p* = 0.3) but not all (for HC-AT: W = 0.86 *p* = 0.005; for HC-VT: W = 0.91 *p* = 0.05; for BD-VT: W = 0.9, *p* = 0.04) conditions within each group. The permutation-based ANOVA on individual just noticeable difference (JND) considering group, condition and age showed a significant main effect of group (F = 4.06, *p* = 0.02) while all the other main effects and interactions were not statistically significant (see Table 1). Specifically, age was not significant (F = 2.5, *p* = 0.1). Post-hoc comparisons between groups confirmed a significant difference between HC and both patients with BD (t = −5.5, *p* < 0.001) and SZ (t = −6.1, *p* < 0.001) but not between the two groups of patients (t = 0.35, *p* = 0.7). Bayes Factors confirmed these results by showing support for the null hypothesis (i.e., µ = 0, groups performed similarly) when comparing BD and SZ (BF10 = 0.32, r = 0.707), and for the alternative hypothesis when comparing HC and BD (BF10 = 6479.08, r = 0.707), and HC and SZ (BF10 = 34645.89, r = 0.707).

**Table 1:** Results of the permutation-based ANOVA for JND and PSE.

	Degrees of freedom for the numerator	Degrees of freedom for the denominator	F-statistic	Resampled *p*-values	Generalized eta squared
**JND:**					
Group	2	56	4.06	0.02	0.1
Age	1	56	2.54	0.1	0.04
Condition	2	112	0.23	0.8	0.004
Group X Age	2	56	1.08	0.4	0.03
Condition X Age	2	112	0.27	0.8	0.005
Group X Condition	4	112	0.82	0.5	0.03
Group X Condition X Age	4	112	0.73	0.6	0.02
**PSE:**					
Group	2	56	0.15	0.9	0.005
Age	1	56	0.08	0.8	0.001
Condition	2	112	0.79	0.4	0.01
Group X Age	2	56	0.5	0.6	0.02
Condition X Age	2	112	0.85	0.4	0.01
Group X Condition	4	112	0.98	0.4	0.03
Group X Condition X Age	4	112	1.26	0.3	0.04

Instead, similar accuracy emerged between groups and conditions, considering age as covariate, from the permutation-based ANOVA on individual point of subjective equality (PSE). No significant main effects or interactions were found (*p* > 0.05, see Table 1). Bayes Factors confirmed the lack of differences across groups and conditions for PSE by showing support in favor of the null hypothesis (i.e., all effects = 0; for Group: BF10 = 0.35; for Condition: BF10 = 0.05; for Group + Condition: BF10 = 0.02; for Group X Condition: BF10 = 0.01). Mean values of PSE for each group and condition are shown in Table 2.

**Table 2:** Mean PSE values for each group and condition.

	AT	AV	VT
**HC**	23.9	−35.07	−25.85
**BD**	−28.08	49.6	−7.68
**SZ**	34.88	7.98	66.27

Subsequent correlational analyses demonstrated a significant association between individual JNDs and the positive subscale of the PANSS (z = 3.3, *p* = 0.003). Instead, individual JNDs were not significantly correlated with scores from PANSS negative subscale (z = 1.3, *p* = 0.7), YMRS (z = 1.7, *p* = 0.3), and HAM-D (z = −0.9, *p* = 0.9)

**Fig. 3:**
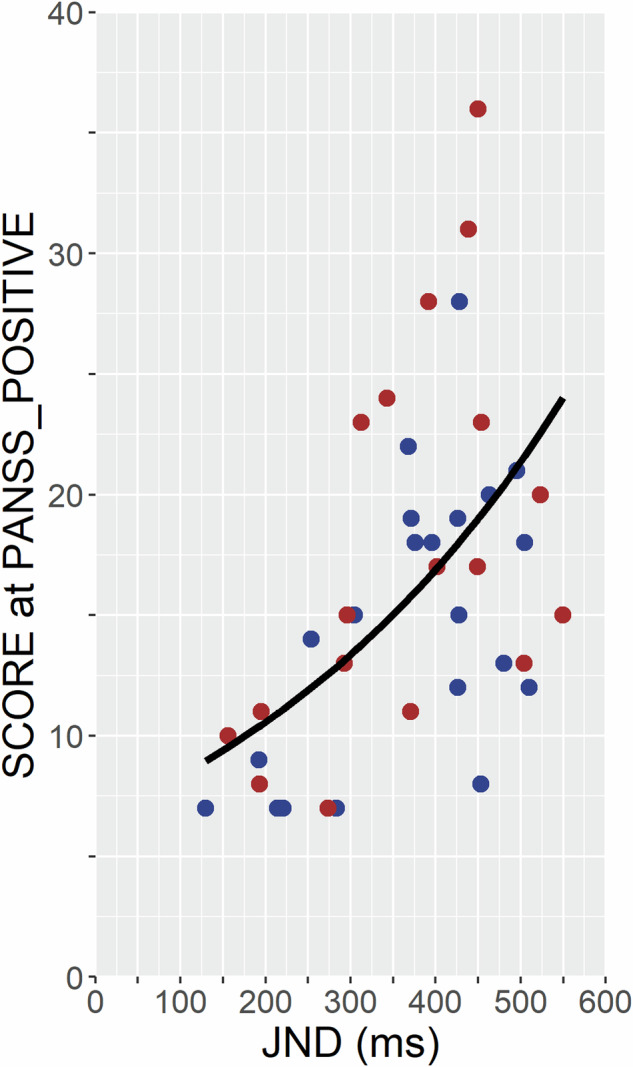
Association between scores on the positive subscale of the PANSS and mean individual JNDs reflecting precision in the TOJ tasks. Results of the negative binomial GLM with log-link function (*glm1*) are plotted. Red dots represent patients with BD; blue dots represent patients with SZ.

Following the outcome of the previous analysis step, we further explored the relationship between temporal precision (JND) and scores on the positive subscale of the PANSS utilizing a negative binomial GLM with logarithm as link function (*glm1: PANSS Positive* ~ *1* + *JNDs*). The model yielded a Nagelkerke’s R-squared of 0.47, signifying that the model explained approximately 47% of the variance in scores at the positive subscale of the PANSS. Moreover, the analysis revealed a significant association between these variables (Estimate = 0.002 ± 0.0005, z = 4.4, *p* < 0.001). This result is shown in Fig. 3.

In the end, Kendall’s tau-b correlational analyses revealed a lack of significant association between medication dosage and temporal precision (for antipsychotics: z = 0.48, *p* = 0.9; for mood stabilizers: z = −0.67, *p* = 0.9; for benzodiazepines: z = −1.4, *p* = 0.6; for antidepressants: z = −0.7, *p* = 0.9).

## Discussion

The present research investigated multisensory temporal coding in patients with SZ, BD and HC to explore a plausible link between alterations in temporal structure of multisensory events and psychopathological dimensions underlying psychosis. The results demonstrated that both SZ and BD patients exhibit poorer precision in temporally ordering sensory stimuli compared to HC, and this impairment is associated with the presence of positive symptomatology. Specifically, patients are less precise in temporally ordering stimuli independently of the sensory modalities involved (audio-visual, visuo-tactile, audio-tactile), and their worst performance is explained by positive symptoms as measured by the positive subscale of the PANSS. Instead, performance is not associated with any of the other subscales of the PANSS related to negative and generalized psychopathological symptoms or depressive and manic symptoms as measured by the HAM-D and the YMRS, respectively.

The worst performance of patients with SZ is in line with previous studies investigating the ability to code events in time within the visual^12–17^, audio-visual^18–21^ and audio-tactile^22^ domains. Our findings confirm results about the audio-visual and audio-tactile stimuli while adding that the deficit also expands to visuo-tactile sensory inputs. Previous research considering unisensory and multisensory stimuli in SZ has already demonstrated that the impairment in multisensory temporal processing goes beyond what can be explained by unisensory dysfunction^18^. The fact that the reduced precision (i.e., higher JND) is not dependent on the sensory modalities involved in the current work highlights even more that the deficit is not intrinsic to a specific sensory system. Still, it rather concerns the overall ability of patients to segment sensory events into separate percepts.

The results about patients with BD can seem contradictory to one previous study assessing temporal order in this population. We observed that patients with BD behaved as SZ patients overall; their precision at the task did not differ from that of patients with SZ, while it was statistically worse than HC. Recently, with a similar task but involving unisensory visual stimuli, Arrouet et al. ^12^ showed that patients with BD were not different compared to SZ patients and HC. However, considering the psychopathological dimension, the other findings can be easily reconciled. While Arrouet et al. ^12^ did not explore the role of the symptomatology, we found that the presence of positive symptoms can explain the patients’ performance. Thus, it could be that patients with BD involved in the previous study had fewer psychotic profiles. Our main finding is indeed that an alteration in multisensory temporal structure is related to specific psychotic symptoms rather than a particular diagnosis.

This hypothesis is supported by other studies reporting a role of psychosis while investigating temporal processing^18,19^. In 2011, Schmidt et al. ^15^ suggested that forthcoming studies should shift their focus from SZ specifically to psychosis in general. This suggestion stemmed from their observation that, regardless of a diagnosis of chronic SZ, individuals who have recently encountered a first psychotic episode required longer temporal intervals compared to HC to identify a temporal asynchrony. Thus, abnormal temporal coding could be a characteristic of psychosis in general and not specific to SZ. In addition, Foucher et al. ^19^ found, in a group of SZ patients, a significant correlation between precision in judging the simultaneity of audio-visual stimuli and disorganization, intended as conceptual disorganization, excitation, difficulties in abstraction, and stereotyped thinking. Interestingly, this association was missing for the unisensory auditory and visual conditions, suggesting its specificity for multisensory temporal processing. Accordingly, other studies also failed to find a significant association between unisensory visual precision and positive symptoms^13,14,16^.

The specific involvement of multisensory processing is in line with previous research showing different patterns of multisensory integration in SZ patients e.g.,^31–35^. Within this context, we found a specific association between multisensory temporal processing and the score on the positive subscale of the PANSS^36^ independently of the diagnosis. It is essential to mention what measures this index. The PANSS is a standardized, clinical interview that rates the presence and severity of positive and negative symptoms, and general psychopathology for people with SZ within the past week. The positive subscale investigates delusions, conceptual disorganization, hallucinatory behavior, excitement, grandiosity, suspiciousness/persecution, and hostility. Interestingly, we observed that performance of patients is predicted by the positive subscale and not by the negative and general psychopathology ones. Additionally, we found that precision was not predicted by scores at the YMRS^37^ and the HAM-D^38^. These two scales are commonly used to address manic symptom severity levels and depressive symptoms. Positive symptoms, such as delusions and hallucinations, and conceptual disorganization are core aspects of psychosis. Showing that they are the only ones associated with multisensory temporal structure independently of the diagnosis has important implications. It has been suggested that combining sensory information that is typically perceived as separate can result in sensory overload, ambiguous perceptual identity, and perception of an improperly filtered, confusing world, which could cascade into self-disturbances^11,39^. Yet, this is only one possible interpretation. Temporal order is also closely related to causality, and may be related to delusions rather than fragmentation and disorganization symptoms. Moreover, in a previous experiment, it was demonstrated that despite difficulties in explicit temporal order judgments, SZ patients subliminally processed stimuli in time similarly to controls. However, unlike HC, they processed stimuli individually rather than sequentially^40,41^. That is, while controls preferentially processed the second of two stimuli, SZ patients preferentially processed the first. The authors interpreted this finding as evidence of disturbed predictive coding in SZ, suggesting a compromised ability to anticipate new events while still focused on current ones. If this is the case, the difficulty in distinguishing when an event happens relative to another may be the cause of the feelings of time discontinuity frequently reported in psychosis at a phenomenological level^42^. Hence, our findings support the hypothesis that low-level temporal processing is related to clinical symptoms like delusions, hallucinations, and conceptual disorganization but future studies are needed to deepen the relationship between temporal processing alterations and specific psychopathological domains. Moreover, the categorial distinction between SZ and BD is controversial^43^. Current biomarkers have proven inadequate in reliably differentiating between SZ, schizoaffective disorder, and BD due to inconsistent results across cognitive, electrophysiological, eye movement, and brain imaging studies^44^. Our findings are consistent with the hypothesis of a continuum between affective and non-affective psychoses and suggest that abnormalities in temporal structure could be an intriguing biomarker. The performance at temporal processing perceptual tasks might be indicative of the severity of psychotic symptoms.

Despite taking several precautions to mitigate the potential impact of cognitive difficulties in patients—such as allowing participants to skip trials and shortening the duration of blocks—we cannot entirely rule out their influence on our results. Previous studies demonstrated that the temporal integration deficit within a 100 ms timescale was independent of attention deployment in SZ patients^45^ and deficits of SZ patients in the temporal order of rapidly successive visual events was independent of any comorbid attentional dysfunction^13^. However, future studies should directly examine the relationship between temporal order processing and attention to clarify the specific role of this cognitive factor. In line with other studies, we did not find significant correlations between temporal precision and medications considering equivalent dosage for antipsychotics, mood stabilizers, benzodiazepines, and antidepressants^13,14,16,19^.

Our study have significant limitations that need to be considered. First, the sample size was relatively small, which may impact the generalizability of our findings. Second, we did not ask HC to complete the psychopathological assessment. Since experiences such as hallucinations and delusions can also occur in the neurotypical population, failing to account for these experiences represents an issue. Future studies should include such assessments to confirm the unique association between positive symptoms and temporal order processing. Additionally, BD patients have higher PANSS positive subscores and higher antipsychotic medication dosages than SZ patients. A possible reason for this result, which seemingly contrasts with the literature e.g.^46^, could be that SZ patients in our study more frequently received long-acting antipsychotic formulations, whereas BD patients often have a recent history of discontinuity in their psychopharmacological therapies before hospitalization. This may result in SZ patients having better pharmacological coverage and fewer acute positive symptoms upon admission. Moreover, our study utilized the QUEST method^47,48^, which has been successfully applied in previous research involving both clinical populations and children (e.g.,^49–51^). However, it has been suggested that JNDs obtained with QUEST might be smaller and potentially less reliable compared to those obtained with traditional methods^52^. Additionally, although the choice of 10 ms stimuli was based on established literature^18,53,54^, it should be noted that this may add complexity to the task, which needs to be taken into account.

To conclude, this research highlights altered temporal ordering of multisensory stimuli among patients with schizophrenia (SZ) and bipolar disorder (BD), closely associated with their positive symptoms. These findings suggest that abnormalities in multisensory temporal processing may play a crucial role in the underlying mechanisms of psychosis. This has significant implications for screening and intervention strategies, where tasks and training focused on temporal processing could be effectively utilized to address psychotic symptoms.

## Methods

### Participants

22 patients with Schizophrenia (SZ), 21 patients with Bipolar Disorder (BD), and 21 healthy controls (HC) took part in the study. Participants were admitted to the Psychiatric Clinic of the IRCCS Ospedale Policlinico San Martino, Genoa, Italy. The study was approved by the local Ethical Committee, and all the participants gave their written informed consent. Inclusion criteria were the following: diagnosis of SZ or BD according to the Diagnostic and Statistical Manual for Mental Disorders-Fifth Edition (DSM-5) criteria^55^. Exclusion criteria included the following: psychiatric disorders other than SZ or BD, history of drug and alcohol addiction, neurological disorders, and severe somatic diseases. Subjects details are provided in Table 3. All healthy individuals had no history of psychiatric, neurological, or cognitive disorders. All participants were native Italian speakers.

**Table 3:** Participants’ demographic and clinical characteristics.

	HC	BD	SZ
Sample size	21	20	21
Age (mean ± SD)	41 ± 9.9	44.9 ± 15.2	44.9 ± 14.2
Male/Female	8/13	7/13	6/15
Years of disease duration (mean ± SD)	-	18.3 ± 13.9^a^	17.5 ± 11.2
HAM-D total score (mean ± SD)	-	10.1 ± 5.5	11.5 ± 5.8
YMRS total score (mean ± SD)	-	13.7 ± 9.5	9.1 ± 7.6
PANSS Negative Scale total score (mean ± SD)	-	11.1 ± 8.6	17 ± 8.4
PANSS Positive Scale total score (mean ± SD)	-	17.2 ± 8.1	14.7 ± 5.9
PANSS General Psychopathology Scale (mean ± SD)		35.2 ± 9.9	34.8 ± 9.8
Antipsychotic treatment equivalent mg/day (mean ± SD)	-	510.3 ± 951.5^b^	293.5 ± 249.6
Mood stabilizer treatment equivalent mg/day (mean ± SD)	-	999.4 ± 571^b^	580.2 ± 525.9
Benzodiazepine treatment equivalent mg/day (mean ± SD)	-	7.6 ± 9.8^b^	6.1 ± 8.3
Antidepressant treatment equivalent mg/day (mean ± SD)	-	19.7 ± 47.8^b^	30.4 ± 92.5

*SD* standard deviation, *HC* healthy controls, *BD* patients with bipolar disorder, *SZ* patients with Schizophrenia, *HAM-D* Hamilton Depression Rating Scale, *YMRS* Young Mania Rating Scale, *PANSS* Positive and Negative Syndrome Scale

^a^Information is missing for one participants

^b^Information is missing for four participants

Three participants have been excluded from the analyses because they were identified as outliers (the Interquartile Range method was to address outliers in temporal precision within each group and task). Thus, the remaining participants consisted of 21 SZ patients (mean age ± standard deviation = 44.9 ± 14.2 years old, female = 6), 20 BD patients (44.9 ± 15.2 years old, female = 7), and 20 healthy controls (41 ± 9.9 years old, female = 8). Age was not significantly different across groups (F_2,59_ = 0.5, *p* > 0.05, ges = 0.02). Among the participants in the BD group, 10 individuals were experiencing a depressive episode, 7 individuals exhibited a manic state, and 3 individuals had a mixed episode at the time of testing.

### Apparatus and stimuli

**Fig. 4:**
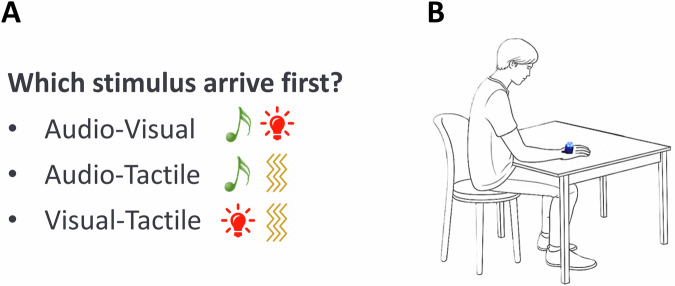
Temporal Order Judgment Task (TOJ). **A** In each block (audio-visual, audio-tactile, or visuo-tactile stimuli), participants were asked to discriminate which stimulus was presented first. **B** Graphical representation of setup, showing the device delivering the stimulation placed on the dominant hand.

Visual, auditory, and vibrotactile stimuli were delivered by a custom-built serial-controlled stimulator 20 mm × 20 mm fixed on the back of the dominant hand using transparent film, which did not impact the stimulations (^56^ see Fig. 4, right panel). The visual stimulus consisted of a high brightness blue LED with a diameter of 1.5 cm, lasting for 10 ms. The vibrotactile stimulus was a 10 ms continuous pure vibration (112 Hz). The auditory stimulus was a 10 ms 926 Hz pure tone pulsed at a frequency of 5 Hz. All stimuli were generated and temporally controlled by MATLAB^57^ with the Psychtoolbox-3 package^58–60^. All timings were verified with an oscilloscope prior to testing.

### Design

Participants performed a Temporal Order Judgment task (TOJ). They were presented with pairs of audio-tactile (AT), visuo-tactile (VT), or audio-visual (AV) stimuli with different stimulus onset asynchronies (SOAs) and were instructed to report which stimulus appeared first. Each condition (AT, VT, AV) was presented in a separate block of 52 trials. The order of blocks was randomized between subjects to control for and avoid an effect of order. For each trial within a block, the SOA was determined adaptively by a QUEST routine on a trial-by-trial basis. QUEST is an adaptive algorithm that uses a Bayesian approach to set the SOA using all the information available from previous trials, supplemented by prior knowledge from the literature^47,48^. The SOA ranged from 0 ms to 800 ms.

### Experimental procedure

The participant sat at a desk with the dominant hand on the table and the multisensory stimulator on the back of the hand (Fig. 4). The distance of the stimulator from the eyes was 50 cm. The experimenter sat at the computer keyboard to the right side of the participant. All participants were instructed to maintain a stable head position. However, head and body orientation were continuously monitored during the experiment by the experimenter. Responses were given orally and the experimenter keyed the responses into the computer manually and initiated the next trial. Maximum response time was set to 10 s for trial, after that the next trial occurred. Since the stimuli had a short duration, to avoid random responses and partially account for potential attentional lapses, we provided participants with the option to skip a trial if they did not perceive it. No feedback was given during the experimental session.

To ensure participants understood the instructions correctly, they were presented with and asked to orally respond to 3-10 simplified trials (SOA = 600-800 ms) before starting the experiment. The experiment began only after the experimenter confirmed that participants correctly answered at least three consecutive trials. Feedback was provided during this instruction phase. There was no formal training session before the experiment. Participants were encouraged to take a break between blocks. Not including breaks, each block took approximately 4 minutes to be completed.

### Psychopathological scales

Different psychopathological scales have been administered to explore symptomatology of patients at the moment of testing: the Hamilton Depression Rating Scale (HAM-D)^38^, the Young Mania Rating Scale (YMRS)^37^, and the Positive and Negative Syndrome Scale (PANSS^36^. The HAM-D is a clinician-administered assessment tool widely used to measure the severity of depressive symptoms. It consists of 21 items covering a range of symptoms such as mood, guilt, sleep disturbances, and suicidal thoughts. Clinicians rate each item on a scale of 0 to 4 or 0 to 2 based on the patient’s reported experiences during the evaluation. Higher scores reflect more severe depressive symptoms. The YMRS is a diagnostic scale designed to evaluate the severity of manic symptoms. It consists of 11 items that assess various aspects of mania, including elevated mood, increased energy, irritability, and disruptive behavior. Each item is scored on a scale ranging from 0 to 4 or 0 to 8, with higher scores indicating more severe manic symptoms. The PANSS is a standardized assessment tool typically used in psychiatry to evaluate the severity of symptoms in individuals with schizophrenia. It comprises 30 items that assess various aspects of psychotic disorders. These items are divided into three subscales: the positive subscale (including positive symptoms such as hallucinations and delusions), the negative subscale (including negative symptoms such as blunted affect and social withdrawal), and the general psychopathology subscale (including anxiety, depression, and disorientation). Each item is rated on a scale from 1 to 7, with higher scores indicating more severe symptomatology.

### Statistical analyses

All analyses were conducted using R^61^. First of all, for each participant and condition (AT, VT, AV), the proportion of trials where one stimulus was perceived as occurring before the other stimulus was plotted as a function of SOA and fitted with a cumulative gaussian function. Following standard psychophysical procedure, the mean and the standard deviation of the best fitting function were obtained as estimates of point of subjective equality (PSE) and just noticeable difference (JND). The PSE represented a participant’s perceptual bias in perceiving synchrony and reflects the accuracy in judgments. The JND represented the smallest temporal difference between the two sensory signals that an individual could detect, it is an index of precision calculated as follows: JND = (x_.75_ − x_.25_)/2, with x_.75_ and x_.25_ corresponding to the stimulus values at which the probability of a given response (e.g., “Audio first”) is equal to 75% and 25%, respectively.

Subsequently, statistical analyses were performed to explore differences between groups in temporal precision (JND). Normality of this variable for each group and condition was checked with Shapiro-Wilk Normality tests. A permutation-based analysis of variance (ANOVA) was then employed to analyze the relationship between groups (HC, BD, SZ), conditions (AT, VT, AV) and age on the JND. Post-hoc comparisons between groups were conducted with permutation-based t-tests, correcting data for multiple comparisons using the Bonferroni correction method. The same approach has been used to assess differences in PSE across groups and conditions taking into account age. Results of JND and PSE across groups and conditions were further investigated with Bayes Factors.

To address the relationships between temporal precision and the psychopathological dimensions, correlational analyses between the JNDs and the clinical scales (scores at the negative and positive subscales of the PANSS, at the YMRS and the HAM-D) were run using Kendall’s tau-b as correlation metric. Since experimental condition (AT, VT, AV) was not found to be a significant factor in previous analyses, the mean JND value across the three conditions was considered for each participant. Since the score on the positive subscale of the PANSS was significantly correlated, we directly addressed the relationship between this variable and temporal precision. Specifically, a negative binomial GLM with log-link function (*glm1*) was tested with score the positive subscale of PANSS as dependent variable and temporal precision as predictor. In Wilkinson’s notation^62^, the model is described by the formula: ‘Scores at PANSS Positive ~ 1 + JNDs’. Normality of residuals was assessed with Q-Q plots and Shapiro-Wilk Normality tests.

To check for possible confounding effects associated with medications, Kendall’s tau-b correlations were conducted between temporal precision (i.e., mean JND value) and treatment equivalents (mean mg/day) for antipsychotics, mood stabilizers, benzodiazepines, and antidepressants. Results of all correlational analyses were corrected for multiple comparisons with Bonferroni method.

## Data Availability

The dataset presented in this study can be found in online Zenodo repository at the following link: 10.5281/zenodo.13832068.
